# In Situ Construction of High Strength Chitosan/Polydopamine Hydrogel in Alkali System with Potential Radical Scavenging and Bacterial Inhibition

**DOI:** 10.3390/polym18161932

**Published:** 2026-08-07

**Authors:** Zhiwei Jiang, Xiang Xiao, Meng Yu, Qiyang Wang, Ping Fang

**Affiliations:** 1 China School of Environmental Science and Engineering, Sun Yat-Sen University, Guangzhou 510275, China; 2 Key Laboratory of Advanced Energy Materials Chemistry (Ministry of Education), Nankai University, Tianjin 300071, China; 3 The Key Laboratory of Water and Air Pollution Control of Guangdong Province, Guangzhou 510655, Chinafangping@scies.org (P.F.); 4 School of Chemistry and Chemical Engineering, Anhui University, Hefei 230601, China

**Keywords:** chitosan, polydopamine, hybrid hydrogel, alkali aqueous solution, antimicrobial activity, antioxidant property

## Abstract

Herein, a novel chitosan-polydopamine hydrogel was constructed through the in situ self-polymerization of dopamine in a chitosan/alkali aqueous solution. Solid-state ^13^C NMR, FTIR, and SEM confirmed that the formed polydopamine (PDA) particles easily wrapped on chitosan chains to fabricate homogeneous porous hydrogels with pearl-like PDA-chitosan nanofibrils. Thus, the hybrid hydrogels exhibited homogeneous architecture with a high compressive strength of 0.43 MPa. At elevated dopamine (DA) content, the size of PDA particles gradually enlarged from 14 to 32 nm, and a denser network structure was formed because chitosan and DA were partially crosslinked by epichlorohydrin (ECH). Furthermore, PDA endowed the hybrid hydrogels with excellent drug-loading capacity, radical scavenging, and antibacterial activities, which displayed great potential for biomedical and therapeutic applications. Therefore, we provide a new avenue for the creation of polymeric hybrid biomaterials from an alkali aqueous solution.

## 1. Introduction

Recently, biopolymer-based hydrogels have attained considerable attention for biomedical applications due to their inherent biocompatibility, biodegradability, and non-toxicity [1,2,3]. Chitosan is a natural amino polysaccharide derived from the complete or partial deacetylation of chitin [4,5]. Based on chitosan’s intrinsic properties, chitosan hydrogels have potential applications in diverse areas such as wound dressing [6,7,8], food industry [9,10], water treatment [11], and tissue engineering [12]. However, the poor mechanical properties of chitosan hydrogels obtained from their acidic aqueous solutions seriously hinder their practical applications. Therefore, significant efforts should be dedicated to exploring a novel approach for fabricating chitosan-based hydrogels with high mechanical strength [13,14,15].

Various strategies, including crosslinking, nanomaterial reinforcement, and polymer blending, have been developed to enhance the mechanical strength of chitosan hydrogels [16,17,18,19]. Recently, a novel solvent (an alkali/urea aqueous solution) has been demonstrated to effectively dissolve chitosan via a freeze-thaw treatment. In an alkali aqueous solution, chitosan interacts with solvent molecules to form hydrogen-bonded complexes and maintains a stiff chain conformation. During the gelation process, the chitosan chains underwent parallel aggregation to form compact bundles, followed by physical gelation through rapid chain entanglement and crosslinking, resulting in an entirely homogeneous network and enhanced mechanical strength [20]. In addition, another kind of tough chitosan hydrogel was prepared through the deacetylation of chitin hydrogels under alkali conditions [21]. However, chitosan with limited microorganism adsorption function still restricts its further applications. Therefore, considerable endeavors have been devoted to constructing multiple functional chitosan-based hydrogels without depressing their desirable properties.

Dopamine, a catechol-containing biomolecule, can be oxidized and spontaneously self-polymerize in an aerobic atmosphere under alkali conditions to form polydopamine (PDA) with catechol and amine functional groups [22,23]. PDA has received considerable attention owing to its versatile functionalities and excellent adhesive properties toward various material surfaces, which can be used variously to construct diverse coating materials [24,25,26,27]. Moreover, PDA, a melanin-like material, exhibits distinctive biocompatibility, attracting massive interest for various types of biological applications such as antibacterial, drug delivery, and biological imaging [28,29,30]. In virtue of its biological activity, PDA can act as a reducing agent for the in situ reduction of various metal ions and an antioxidant to scavenge active free radicals [31,32]. Specifically, PDA-based hybrid hydrogels with multifunction have been fabricated, in which PDA acts as an antioxidant and provides self-adhesiveness [33]. The obtained multifunctional hydrogels seem to be promising for biomedical and therapeutic applications, including electronic skins, bioelectrodes, and wound dressings [33,34,35,36]. However, convectional PDA-based hydrogels with low mechanical strength hinder their extensive biomedical applications. Therefore, the development of multifunctional hydrogels integrating excellent mechanical properties, biocompatibility, and antibacterial activities is highly desirable.

In this study, a novel chitosan-polydopamine hydrogel with high strength was constructed through in situ self-polymerization of dopamine in chitosan/alkali aqueous solution. Their microstructure, mechanical performance and pH-sensitive characteristics were investigated. Furthermore, cell adhesion and spreading behaviors on chitosan/PDA hybrid hydrogels were conducted to assess their potential for biomedical applications. The hybrid hydrogel also showed excellent antioxidant and antibacterial abilities for potential wound healing or tissue engineering materials. This work will provide a novel perspective on the fabrication of polymeric hybrid biomaterials by an in situ self-polymerization method in an alkali aqueous solution.

## 2. Materials and Methods

### 2.1. Materials

Commercial-grade chitosan, potassium hydroxide (KOH), lithium hydroxide (LiOH), urea, and epichlorohydrin (ECH) were purchased from Sinopharm Chemical Reagent Co., Ltd. (Shanghai, China). The chitosan possessed a viscosity-average molecular weight of 600,000 g/mol and a degree of deacetylation (DD) of 85.1%, which were determined by potentiometry according to the literature method [37]. Dopamine hydrochloride (DA) (99%) and doxorubicin hydrochloride (Dox) were purchased from Aladdin (Shanghai, China). All reagents were used directly as received without any further purification.

### 2.2. Preparation of the Chitosan-PDA Hydrogel

A chitosan solution with a concentration of 5 wt% in LiOH/KOH/urea aqueous solution was obtained according to Duan’s method [20]. The Chitosan-PDA hydrogel was prepared by incorporating dopamine into the alkali chitosan solution through self-polymerization under an ambient environment and using ECH as a cross-linker. In detail, the desired weight of dopamine hydrochloride and 0.5 mL of ECH were added to 30 g of the chitosan solution and stirred at 0~5 °C for 30 min to achieve a brown pregel solution. After centrifugation at 7000 rpm for 5 min to remove entrapped air bubbles, the chitosan-PDA pregel solution was cast into a mold and refrigerated overnight to yield a “raw” chemically crosslinked hydrogel with low mechanical strength. The obtained chitosan-PDA hybrid hydrogel was then immersed in a 75% (*v*/*v*) aqueous ethanol solution for 24 h to terminate the chemical crosslinking reaction. Following extensive washing with deionized water for the removal of residual alkali and urea, the chitosan-PDA hydrogel was achieved. A range of chitosan-PDA hydrogels denoted as CPDA-1, CPDA-2, and CPDA-3, corresponding to the weight of DA of 0.3, 0.6, and 0.9 g, respectively. The reference sample of chitosan hydrogel was prepared using the above procedure without DA and was denoted as CS.

### 2.3. Characterization

The Fourier transform infrared (FTIR) spectra were collected in the range of 4000–400 cm^−1^ using an infrared spectrometer (Perkin Elmer Spectrum Nexus 470, Shelton, CT, USA). The CS and CPDA hybrid hydrogels were first placed in a lyophilizer and subsequently dried at 80 °C under vacuum to remove residual water. Solid-state ^13^C NMR spectra of CS and CPDA hybrid powders were recorded on a Bruker AVANCE III 400 spectrometer (^13^C frequency = 100.12 MHz) using a cross-polarization/magnetic angle spinning (CP/MAS) probe at room temperature.

The structure and morphology of CS and CPDA hybrid hydrogels were characterized by scanning electron microscopy (SEM) and wide-angle X-ray diffraction (XRD). SEM observations of the inner structure of CS and CPDA hydrogels were carried out on a JEOL SU8010 microscope. XRD measurements were operated on an X-ray diffractometer (Bruker, D8 Advance, Karlsruhe, Germany). The XRD patterns were collected using Cu Kα radiation (λ = 0.15406 nm) at 40 kV and 30 mA over the 2θ range from 5 to 40°. The samples were dried at 80 °C under vacuum for 24 h and then ground into powder. The crystallinity index (CrI) of the CS and CPDA powders was calculated using the following equation

(1) $$CrI=\frac{I_{110}-I_{am}}{I_{110}}\times 100\%$$

where *I*_110_ is the maximum intensity of the (110) lattice diffraction peak at 2θ = 20°, and *I*_am_ is the intensity of the amorphous region at 2θ = 16° for chitosan. The mechanical properties of the CS and CPDA hydrogels were evaluated by compression testing using a universal testing machine (AI-7000M, Qingdao, China) at a speed of 1 mm min^−1^. The experiments were conducted with a minimum of three repetitions at room temperature. All experiments were conducted in triplicate at room temperature.

### 2.4. Cell Culture

HeLa cells and Esophageal cancer cells (ECA109) were provided by Nanjing KeyGen Biotech. Co., Ltd. (Nanjing, China) and cultured in high-glucose Dulbecco’s Modified Eagle’s Medium (DMEM) supplemented with 10% fetal calf serum (FCS) and 80 U mL^−1^ of streptomycin. The materials were sterilized by immersion in ethanol followed by UV irradiation. ECA109 cells (10 × 10^4^ cells per well) were seeded onto the material placed in 24-well plates or not seeded onto the material as a control. After a 24 h incubation in a humidified atmosphere (5% CO_2_ at 37 °C), the cells on the substrates were stained with Calcein-AM and subsequently visualized via fluorescence microscope. Each experiment was conducted in triplicate.

### 2.5. pH-Sensitive Swelling Behaviors

To determine the swelling ratios (SR) of the hydrogels, gravimetric measurements were performed in a 2 wt% acetic acid aqueous solution and hydrochloric acid aqueous solutions with varying pH values (ionic strength maintained at 0.1 M via NaCl adjustment). Each experiment was conducted in triplicate. SR was determined as follows:

(2) $$SR=\frac{W_{s}-W_{d}}{W_{d}}\times 100\%$$

where *W*_s_ and *W*_d_ represent the weight of the swollen hydrogel and the dried gel, respectively.

### 2.6. In Vitro Drug Loading and Release Studies

3 mg of dried CS, CPDA-1, CPDA-2, and CPDA-3 particles were immersed in a 1 mg mL^−1^ Dox aqueous solution in the dark for 48 h at 37 °C. The amount of free drug in the supernatant was subtracted from the initial total drug payload to evaluate the drug loading and entrapment efficiency of hydrogels. Subsequently, drug-loaded CPDA-2 particles were immersed in 6 mL of hydrochloric acid aqueous medium with varying pH values (ionic strength maintained at 0.1 M via NaCl adjustment) under constant shaking at 37 °C. At each sampling interval, 2 mL of the release medium was removed for analysis and immediately replenished with an equivalent volume of fresh buffer. The cumulative release amount was determined by calculating the drug amount in each of the release medium. Finally, Dox concentration was quantified via UV-vis spectrophotometry at 232 nm. Each experiment was conducted in triplicate.

### 2.7. Assay for Free Radical Scavenging Activity

The potential antioxidant activity of the CPDA hydride hydrogels was determined using 2,2-diphenyl-1-picrylhydrazyl (DPPH) radical-scavenging assay according to a previously reported method [38]. Briefly, a 0.125 mM DPPH solution was freshly prepared in ethanol prior to use. Then, chitosan/PDA hybrid hydrogels (1 g) were immersed in 5 mL of DPPH/ethanol solution and incubated in the dark for a specified time. The absorbance at 516 nm for DPPH was determined utilizing a UV-vis spectrophotometer (UV-2550, Shimadzu, Kyoto, Japan). Each experiment was conducted in triplicate. The free radical scavenging rate (SR_FR_)was calculated as follows:

(3) $${SR}_{FR}=\frac{A_{0}-A_{i}}{A_{0}}\times 100\%$$

where, A_0_ and A_i_ represent the absorbance at 516 nm for DPPH for the control solution and the experimental solution at different times, respectively.

### 2.8. Antibacterial Activity Tests

Nutrient agar containing 3 g L^−1^ beef extract, 10 g L^−1^ peptone, 5 g L^−1^ NaCl, and 15 g L^−1^ agar was used as the culture medium for *Escherichia coli* (*E. coli*) and *Staphylococcus aureus* (*S. aureus*). All media and materials were sterilized by autoclaving at 121 °C for 2 h prior to use. The agar plates were inoculated with 100 μL of bacterial suspensions (1 × 10^6^ CFU mL^−1^). The hydrogels were cut into discs (6 mm in diameter), followed by incubation at 37 °C for 24 h. Antibacterial activity was evaluated by measuring the inhibition zones.

## 3. Results and Discussion

### 3.1. Construction and Interaction of Chitosan/PDA Hydrogels

**Scheme 1:**
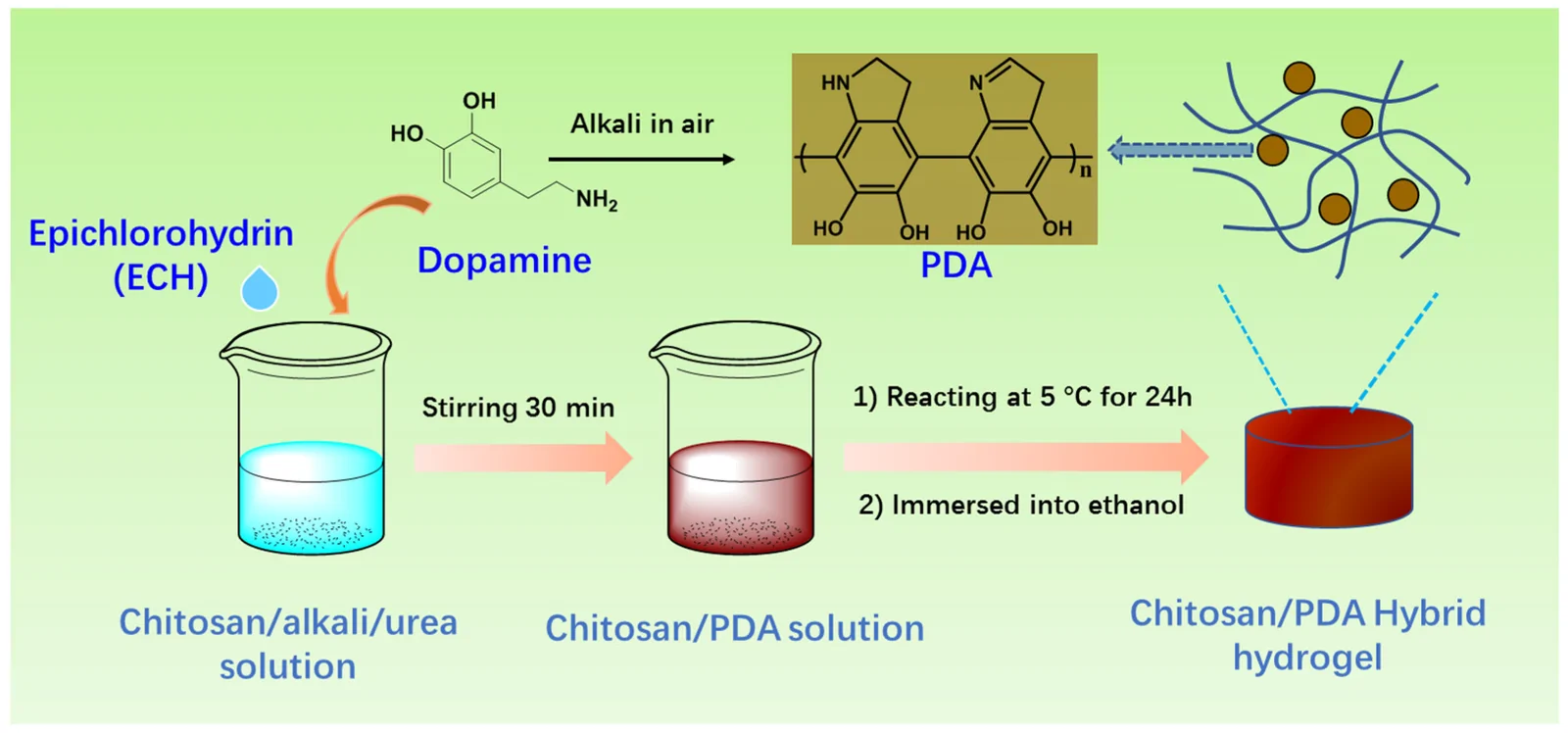
Schematic diagram illustrating the preparation process of the chitosan/PDA hybrid hydrogel.

Here, we designed a novel hybrid hydrogel composed of chitosan and PDA (polydopamine) fabricated in LiOH/KOH/urea aqueous solution (Scheme 1). In brief, dopamine hydrochloride was added to an alkali chitosan solution containing epichlorohydrin under vigorous stirring; the colorless solution rapidly turned pale yellow and subsequently gradually changed to brown. The color change can be explained by the self-polymerized of dopamine hydrochloride under alkaline conditions in an aerobic atmosphere to generate brown polydopamine. The brown solution was successively cast into a mold and refrigerated overnight to form a hydrogel. After immersing for 24 h in a 75% ethanol aqueous solution, the obtained hydrogels were extensively washed with deionized water to remove residual alkali and urea to achieve the chitosan-PDA hydrogels.

**Figure 1:**
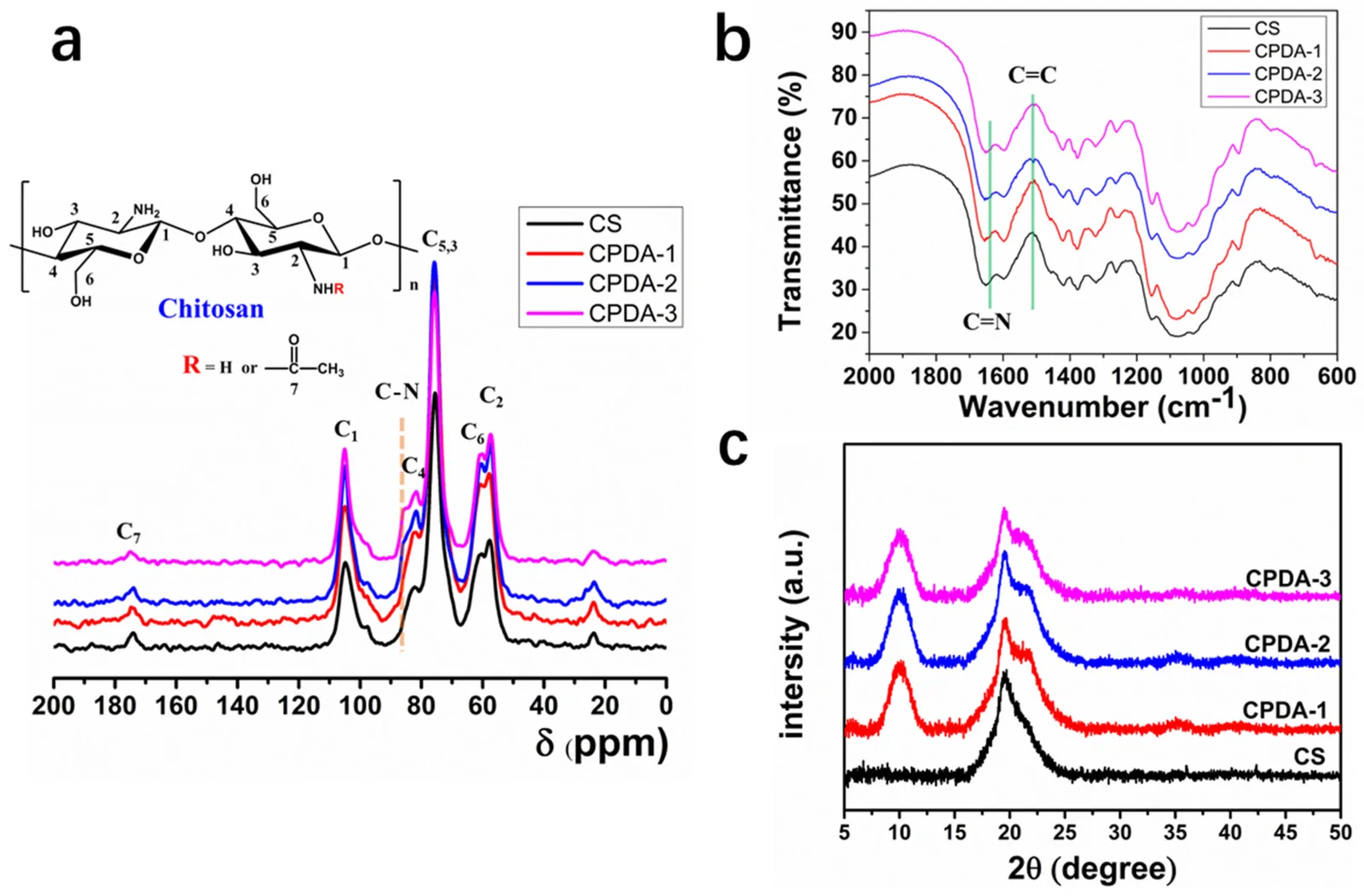
Solid-state ^13^C NMR spectra (**a**), FTIR (**b**), and WAXD (**c**) of CS and CPDA hydrogels.

In order to clarify the interaction of the chitosan-PDA hydrogels, CP/MAS ^13^C NMR spectra of the CPDA and CS hydrogels were carried out, as shown in Figure 1a. In Figure 1a, the CP/MAS ^13^C NMR spectrum of CS hydrogel displays seven main signals. The weak peaks at 23.7 and 174.4 ppm are attributed to the methyl carbons and carbonyl, respectively. The signals at 104.9, 82.2, 75.5, 60.5, and 58.0 ppm are assigned to C_1_, C_4_, C_3_/C_5_, C_6_, and C_2_ of the D-glucosamine units in chitosan, respectively. The signals in solid-state ^13^C NMR for the CS hydrogel were similar to those of the chitin hydrogel, suggesting that chitosan still remains in a rigid-chain conformation in alkaline solution [39]. For CPDA hydrogels, the chemical shifts of the carbon atoms of chitosan stayed basically unchanged except for the resonance signal of C_2_ bonded to NH_2_. A high-field shift of C_2_ in the ^13^C NMR of CPDA-1, CPDA-2, and CPDA-3 hydrogels could possibly be attributed to the formation of hydrogen bonding interactions between the NH_2_ of chitosan and PDA, which increased electron shielding around C_2_ atoms. It was not hard to understand that the formation of PDA did not affect the stiff chain conformation of chitosan in CPDA hydrogels, and PDA just attached to the surface of chitosan chains. In addition, two new observable peaks at 100.2 and 85.6 ppm in the CP/MAS ^13^C NMR spectra of CPDA-1, CPDA-2, and CPDA-3 gels could be assigned to C=C bonds in aromatic rings and C-N bonds obtained through covalent reaction between PDA and chitosan, respectively [40]. This result revealed that PDA was successfully obtained in chitosan alkali aqueous solutions and that the formation of PDA did not change the structure of chitosan. PDA with catechol and amino functional groups can be combined with the organic surface through a Michael addition reaction with an amino or thiol group. Chitosan, with abundant amino groups, can react with PDA; thus, covalent interactions between chitosan and PDA could exist in CPDA hydrogels. The peak strength of C-N bonds in CPDA hydrogels gradually increases at elevated DA content, suggesting an increase in the formed PDA or an enhancement of cross-linking between DA and chitosan by epichlorohydrin.

The functional groups of the chitosan-PDA hydrogels were further characterized via FTIR spectroscopy. Figure 1b and Figure S1 show the FTIR spectra of CS and CPDA hydrogels. The stretching vibration of hydroxyl groups in CS and CPDA hydrogels was located at 3300–3500 cm^−1^, coinciding with the reported data of chitosan [41]. The amide I band of chitosan in CS and CPDA hydrogels appeared as a single peak at approximately 1650 cm^−1^, suggesting that the covalent cross-linking reaction disrupted the intra- and inter-sheet hydrogen bonds within chitosan [21,39]. The characteristic adsorption peak at 1595 cm^−1^ is attributed to the N-H bending vibration of chitosan [21]. Notably, the FTIR spectra of CPDA-1, CPDA-2, and CPDA-3 hydrogels exhibit characteristic chitosan adsorption bands consistent with those of CS hydrogels, revealing that self-polymerization of dopamine in the alkali solution does not affect the chitosan chain conformation, which is consistent with the CP/MAS ^13^C NMR results. In addition, new observable characteristic peaks at 1509 cm^−1^ and 1637 cm^−1^ for all CPDA hydrogels are consistent with C=C and C=N bonds in indole or indoline structures [40], illustrating that PDA could be produced under these synthesis conditions.

**Scheme 2:**
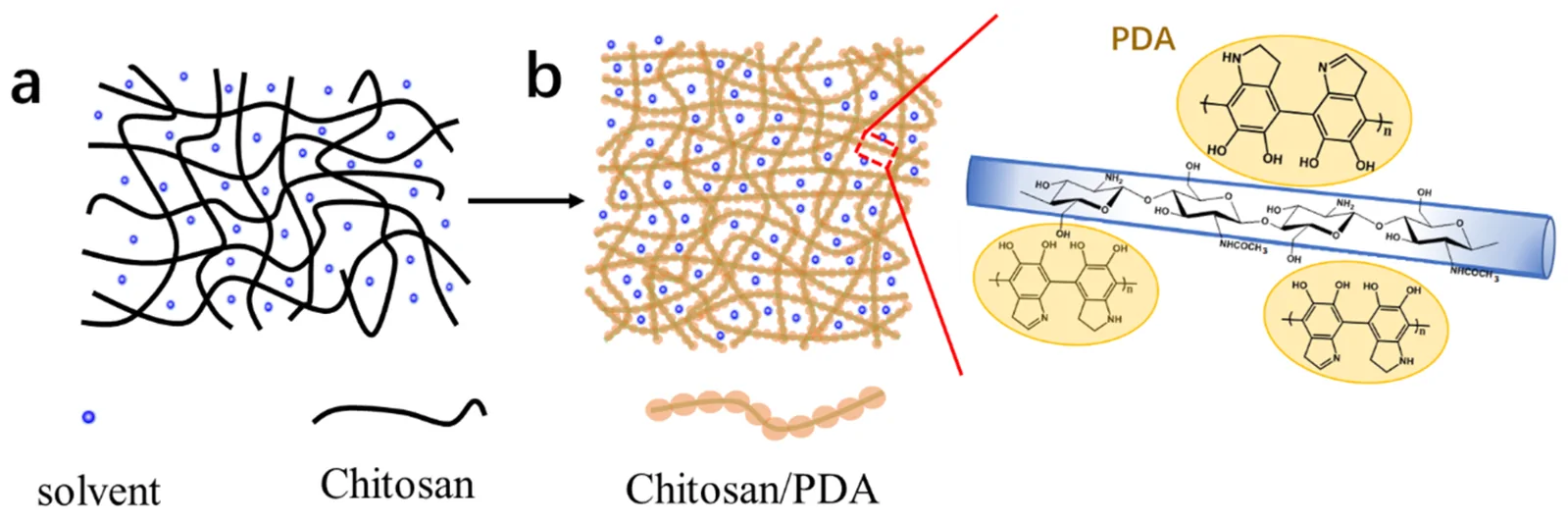
Schematic depiction of CS (**a**) and CPDA (**b**) hydrogels.

XRD patterns of chitosan and chitosan-PDA hydrogels are illustrated in Figure 1c. All samples appeared to have an obvious peak at 19.8°, attributed to (110) lattice diffraction of chitosan [20]. The crystallinity index (CrI) serves as an indicator of the structural regularity within the polymer chain aggregation state. Based on multipeak fitting, the CrI values were 79.2% for CS hydrogel and 71.8–77.2% for the CPDA hydrogels (Table 1). Notably, the CrI of chitosan in CS was higher than that in CPDA, indicating that PDA partially disrupted the ordered packing of the chitosan chains in CPDA. Furthermore, the XRD spectra of CPDA hydrogels showed a broad peak centered at 2θ = 21.5° (*d*-spacing ≈ 4.1 Å), indicating the presence of π-π stacking interaction within PDA particles [42]. This result confirmed that PDA could be fabricated in chitosan alkali aqueous solutions. In view of the above results, a possible schematic depiction of the porous structure with the nanofiber networks in the CPDA-2 gels is proposed in Scheme 2. The introduction of DA into the chitosan/alkali aqueous solution formed CPDA hydrogels with a denser network structure compared with CS hydrogel, suggesting that chitosan and DA were partially chemically cross-linked by ECH.

### 3.2. Morphologies and Mechanical Properties of CPDA Hydrogels

**Figure 2:**
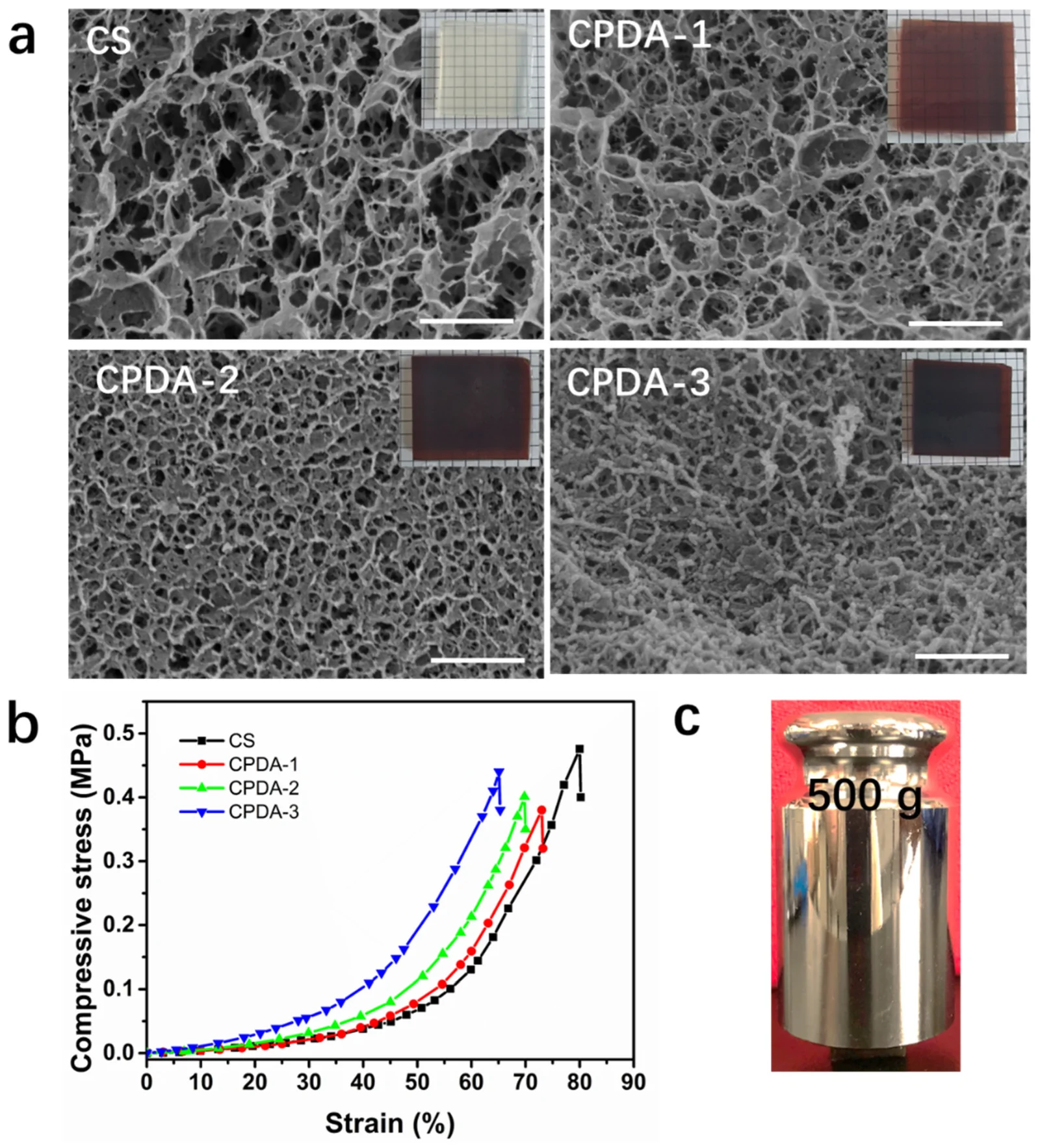
(**a**) SEM images of the cross-sectional structure of CS and CPDA hydrogels; inset: photographs of these hydrogels. Scale bar = 500 nm. (**b**) Compressive stress-strain curves of CS and CPDA hydrogels, (**c**) The picture of CPDA-3 loaded with a 500 g weight.

Figure 2a shows the photographs of the CS, CPDA-1, CPDA-2, and CPDA-3 hydrogels. The CS hydrogel was colorless and transparent, suggesting a homogeneous structure derived from the chemical cross-linking network formed between chitosan and ECH, which is consistent with previous research reported by Duan et al. [20]. The successive oxidation of dopamine in an alkali condition can produce pitchy melanin-like nanoparticles. Thus, the color of the chitosan-PDA hydrogels changed from brown to dark brown with the increase of dopamine content, confirming that PDA was successfully synthesized and had a good composite with chitosan. It was notable that the CPDA-1 hydrogel was transparent, indicating that the formed PDA dispersed well and attached to the chemically cross-linked chitosan networks, resulting in good transmittance of the CPDA-1 hydrogel [33].

SEM analysis can clearly reveal the morphology and network structure of the hydrogel [39]. The cross-section SEM images of the CS and CPDA hydrogels are shown in Figure 2a. All hydrogels displayed a uniform nanofibrous architecture and a well-distributed honeycomb structure with diverse pore sizes (10~200 nm), illustrating certain miscibility between chitosan and PDA particles. Moreover, the chitosan chain networks consisted of nanofibers (23 nm in mean diameter). As expected, the pore size of CPDA was reduced with increasing DA content, revealing that DA may partly participate in covalent reactions to enhance the cross-linking degree. In addition, PDA nanoparticles were adhered uniformly to the surface of chitosan nanofibers, and the intransigent chitosan acted as the main backbone in the CPDA hydrogels, attributed to cross-linking interaction between chitosan and PDA.

It was notable that the size of PDA particles enlarged from 14 to 32 nm in CPDA hydrogels with an increase in dopamine content. The size of PDA particles in CPDA-1, CPDA-2, and CPDA-3 is shown in Figure S2. Furthermore, in CPDA-2 and CPDA-3 hydrogels, PDA nanoparticles guided by chitosan nanofibers as a template were self-assembled to form pearl-like nanofibrils. Table 1 lists the water content values of the CPDA hydrogels. The water content value of CS hydrogel was 92.8% and then decreased gradually from 90.6% to 87.7% with an increase of DA content, further confirming that the structure of CPDA hydrogels was denser than that of CS hydrogel.

As is well known, the mechanical properties of hydrogels are very important for their applications. The compressive stress-strain curves of CS and CPDA hydrogels are illustrated in Figure 2b. Table 1 shows the detailed mechanical parameters of CS and CPDA hydrogels. It can be observed that the CS and CPDA hydrogels exhibited excellent compressive strength with over 0.38 MPa of compressive fracture stress, which is superior to that of chitosan obtained via acetic acid dissolution (0.05 MPa). The compressive mechanical properties of CPDA hydrogels exhibited a similar level to those of CS. It can be explained that the PDA particles were closely attached to the chitosan nanofibers. Therefore, the mechanical strength of the CPDA hydrogels is mainly derived from their strong chitosan backbone network structure [20]. Thus, an increase in dopamine content could not affect the mechanical properties of the CPDA hydrogels. Furthermore, the compressive fracture strains decreased from 80.2 to 68.5% as the dopamine content increased from 0 to 37.5%. This could be explained by the fact that DA may partly participate in the cross-linking reaction, and the pore size was reduced at an elevated dopamine content, leading to a slight decrease in the toughness of CPDA. Furthermore, CPDA-3 can support the weight of 500 g without destruction in Figure 2c. These results revealed that the CPDA hydrogels possessed a homogeneous architecture and strong networks woven with pearl-like nanofibrils through chemical and physical dual cross-linking, which were responsible for their high compressive strain and stress properties.

### 3.3. Biocompatibility and pH Sensitivity of CS and CPDA Hydrogels

**Figure 3:**
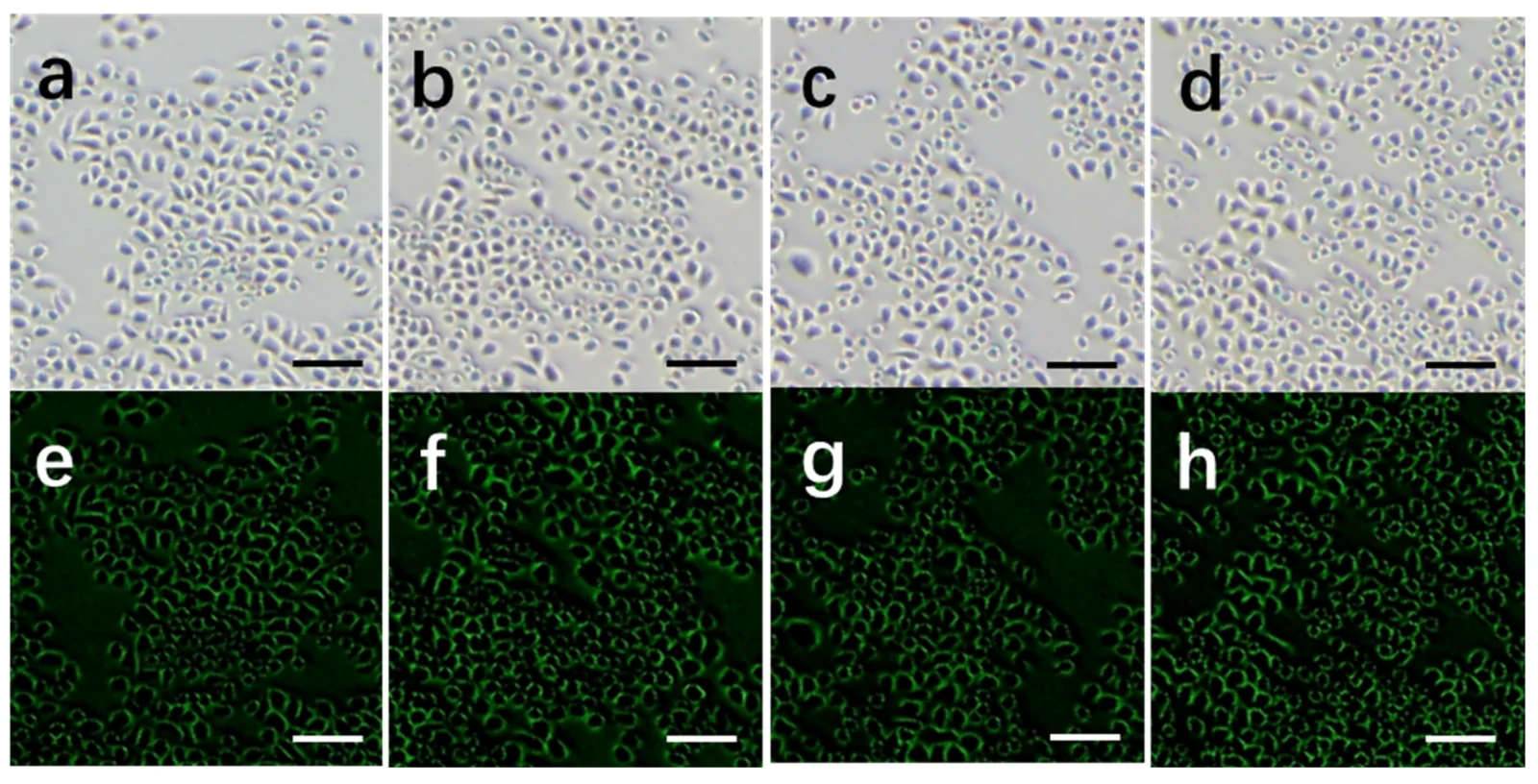
Optical microscopy images and Fluorescent images of ECA 109 cells cultured on the CS (**a**,**e**), CPDA-1 (**b**,**f**), CPDA-2 (**c**,**g**), and CPDA-3 (**d**,**h**) and for 1 day. The scale bar is 100 μm.

PDA and Chitosan show good cell adhesion capacity, antibacterial properties, and non-toxicity [43,44]. It is necessary to estimate the cytocompatibility of the CPDA hybrid hydrogels through ECH cross-linking. The cell viability values for CS and CPDA hydrogels via in vitro cytotoxicity tests are higher than 95% (Figure S3), revealing excellent biocompatibility. Moreover, the honeycomb structure of the CPDA hydrogels was very significant for cell affinity and proliferation. The ECA109 cell was selected to investigate cell growth on CPDA hydrogels and further assess their cytocompatibility. Figure 3 illustrates optical microscopy and fluorescence images of ECA109 cells stained with Calcein-AM, cultured on the surfaces of chitosan gel, CPDA-1, CPDA-2, and CPDA-3 for 1 day. Obviously, the seeded ECA109 cells exhibited good adhesion, spreading, and proliferation on the hydrogels, displaying good cell affinity and biocompatibility. The results confirmed that CPDA composite hydrogels through ECH cross-linking exhibited great potential for biomedical and therapeutic applications.

**Figure 4:**
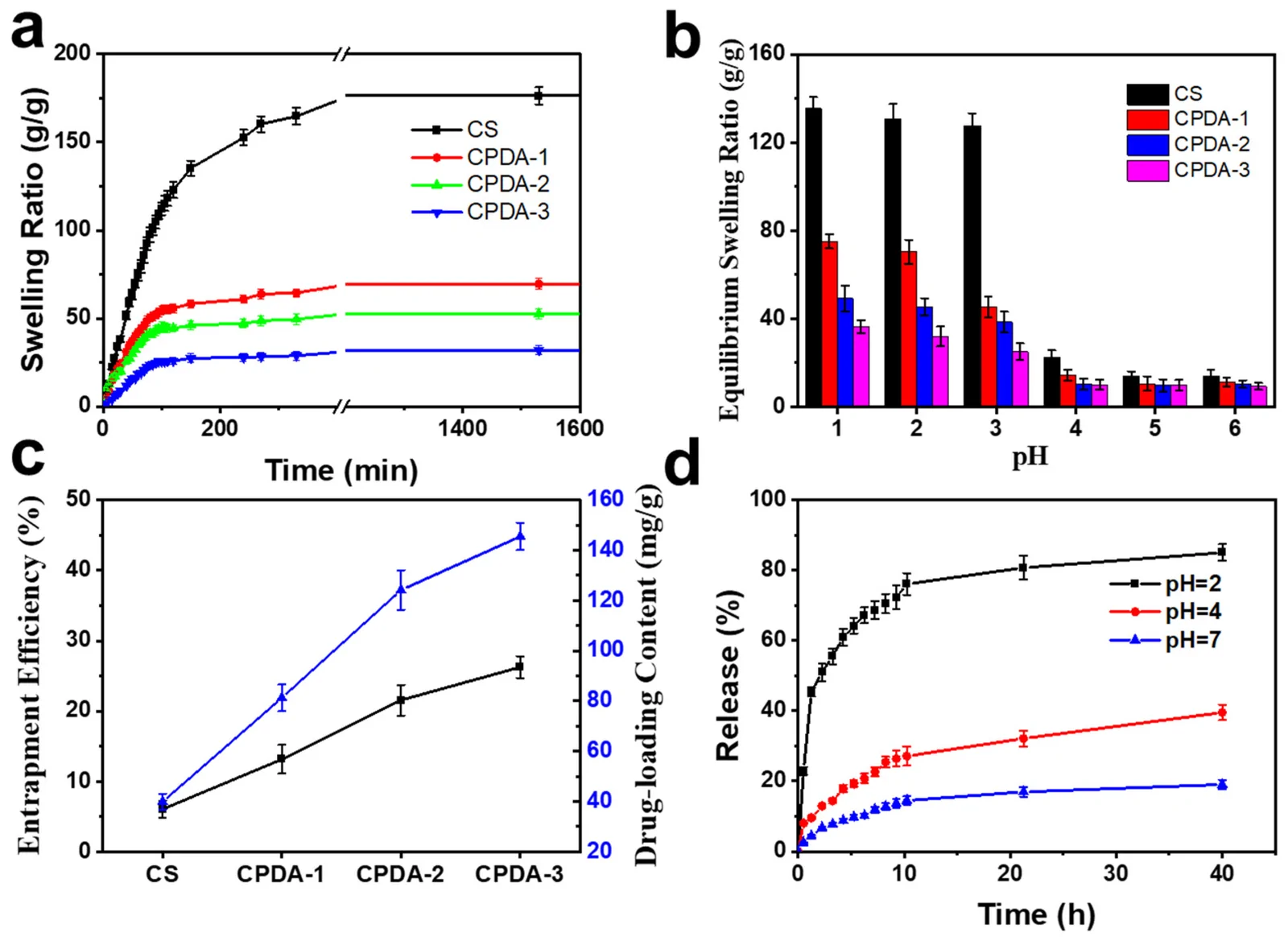
Swelling kinetics of the CS and CPDA hydrogels in 2 wt% acetic acid aqueous solution at 37 °C (**a**). The equilibrium swelling ratio of CS and CPDA hydrogels in hydrochloric acid aqueous solution with different pH values (**b**). Drug-loading content and entrapment efficiency of CS and CPDA hydrogels for Dox with a concentration of 1 g L^−1^ (**c**). The drug release kinetics curve for CPDA-2 at different pH mediums (**d**).

The chitosan-based hydrogels showed well pH-responsive behavior because of the NH_2_ groups in chitosan. Figure 4a illustrates the swelling ratio curves of the CS and CPDA hydrogels in a 2 wt% acetic acid aqueous solution. Obviously, all hydrogels achieved the swelling equilibrium after 8 h of immersion in the aqueous acid solution. The equilibrium swelling ratios of CS, CPDA-1, CPDA-2, and CPDA-3 were decreased from 133 to 73, 43, and 36, respectively. It can be explained that the crosslinking degree of the CPDA hydrogels increased with the PA content, leading to a reduction in the equilibrium swelling ratio. This result declared that PA could partly participate in the crosslinking reaction during the formation of CPDA hydrogels. The effect of pH values on the equilibrium swelling ratio of the chitosan and CPDA hydrogels was also studied, as shown in Figure 4b. When pH < 4, all of the hydrogels exhibited dramatic swelling behavior. It can be inferred that the chitosan chains and PDA bearing positively charged groups are expanded at pH < 4 because of strong electrostatic repulsive interaction between the protonated -NH_3_^+^ groups. Therefore, CPDA hydrogels exhibited obviously pH-sensitive behavior.

Dox is a clinically used chemotherapeutic agent for cancer therapy [45]. Dox was selected as a model drug to investigate the drug loading capacity and release behavior of CPDA hydrogels. Drug-loading content and entrapment efficiency of CS and CPDA hydrogels for 1 g L^−1^ Dox are shown in Figure 4c. The entrapment efficiency increased from 6.1% to 26.3% in CPDA hydrogels as the DA content increased from 0% to 37.5%. Meanwhile, the drug-loading capacity of Dox increased with the DA content in CPDA hydrogels and achieved the maximum value of about 146 mg/g in CPDA-3. The result could be ascribed to the strong hydrogen and π-π stacking interactions between PDA and Dox [46]. The drug-release kinetics curve for CPDA-2 in different pH mediums was also studied, as shown in Figure 4d. The reduction of pH value was useful to release Dox from CPDA-2, but the release rate became low at an elevated pH value from 2 to 7. After 40 h of incubation, the cumulative release of Dox at pH = 2 and 4 was 85% and 40%, respectively, while at pH = 7 it was only 19%, showing a smart controlled release of CPDA for Dox. Therefore, CPDA represents a smart hydrogel with promising potential for biomedical applications.

### 3.4. Antioxidant Property and Antibacterial Activity

**Figure 5:**
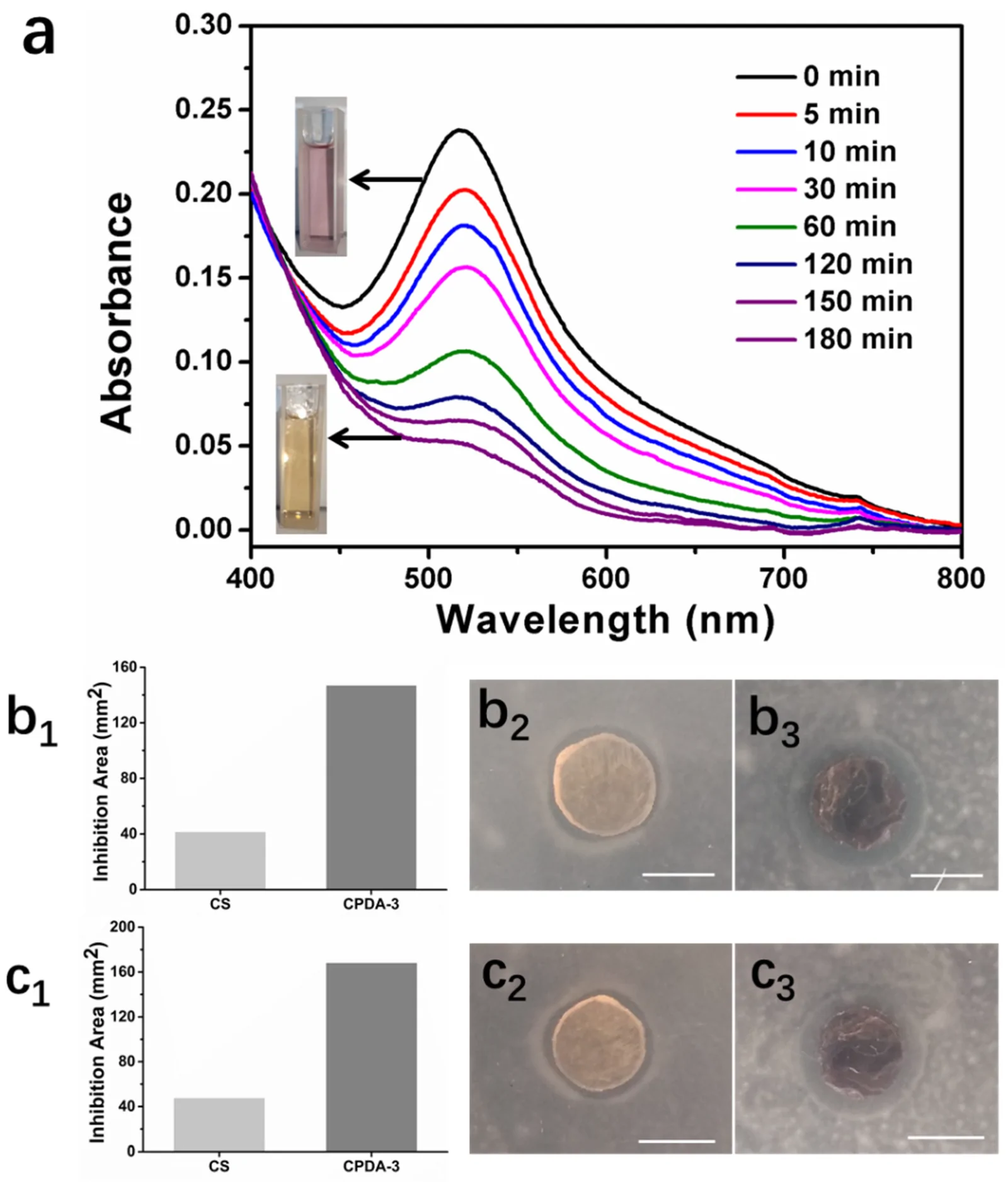
(**a**) DPPH radical scavenging activity of CPDA-3 hybrid hydrogel at different times. (**b**,**c**) Inhibition area of CS and CPDA-3 for *E. coli* (**b_1_**) and *S. aureus* (**c_1_**). Macroscopic images of CS and CPDA-3 inhibition halos in *E. coli* (**b_2_**,**b_3_**) and *S. aureus* (**c_2_**,**c_3_**). The scale bar is 5 mm.

PDA containing catechol groups exhibits excellent antioxidant activity in biological systems, which is beneficial for promoting wound healing [47]. The free radical scavenging activity of CPDA hydrogels was evaluated using the DPPH assay. After 60 min of incubation, the hybrid hydrogels showed over 50% free radical scavenging efficiency (Figure S4). The free radical scavenging rate was found to increase by raising the PDA content in the hybrid hydrogels. After 180 min incubation, nearly 80% of free radicals for CPDA-3 were captured, as shown in Figure 5a, suggesting CPDA hybrid hydrogels possessed excellent antioxidant performance. Thus, the CPDA hybrid hydrogels can be considered for anti-inflammation or wound healing.

Antimicrobial activity of biomaterials plays a significant role in wound healing. As PDA exhibits intrinsic antimicrobial properties, two typical bacteria, Gram-negative *E. coli* and Gram-positive *S. aureus*, were selected as model microorganisms to evaluate the antibacterial activity of CPDA hydrogel. The in vitro antibacterial performance of CPDA-3 was investigated against *E. coli* and *S. aureus* using the disk diffusion method. The inhibition zones induced by CS and CPDA-3 are shown in Figure 5b,c. The CS sample exhibited antibacterial activity against both *E. coli* and *S. aureus*, with inhibition zone areas reaching 147.1 and 168.3 mm^2^, respectively. This was attributed to the antibacterial feature of the NH_2_ group from chitosan. Compared with CS, CPDA-3 hybrid hydrogel demonstrated enhanced antibacterial activity against both *E. coli* and *S. aureus*, with inhibition zone areas reaching 147.1 and 168.3 mm^2^, respectively. The improved antibacterial performance could be attributed to the synergistic antibacterial effect of NH_2_ groups from chitosan and catechol groups from PDA in CPDA-3. These results clearly showed that the CPDA hybrid hydrogels possessed promising potential for wound healing applications.

## 4. Conclusions

In this study, a novel chitosan-polydopamine hybrid hydrogel was constructed successfully through in situ self-polymerization of dopamine in an alkaline chitosan aqueous solution and then a chemical-physical double cross-linking method. XRD, solid-state ^13^C NMR, FTIR, and SEM showed that the novel CPDA hydrogels had homogeneous architecture, and the network was woven with pearl-like chitosan-PDA nanofibrils because the formed PDA particles easily wrapped around chitosan chains. CPDA hydrogels displayed excellent compressive properties, and their compressive fracture stress could be as high as 0.43 MPa. Moreover, the size of PDA particles in CPDA increased from 14 to 32 nm, and a denser honeycomb structure was fabricated with an increase in DA content. Meanwhile, the CPDA hybrid hydrogels exhibited high mechanical properties, well pH-responsive, smart controlled drug-release behavior, biocompatibility, radical scavenging, and antibacterial activities. Therefore, the hydrogel shows great potential for biomedical applications. In addition, this work provides a new strategy for constructing polymeric hybrid biomaterials using an alkaline system.

## Figures and Tables

**Table 1:** Physical and Mechanical Properties of CS and CPDA Hydrogels.

			Compressive Properties	
Sample	Water Content (%)	Cr I ^a^ (%)	σ (MPa)	ε (%)
CS	92.8	79.2	0.47 ± 0.12	80.2 ± 2.5
CPDA-1	90.6	77.6	0.38 ± 0.08	73.1 ± 3.1
CPDA-2	88.5	75.1	0.41 ± 0.15	69.8 ± 1.8
CPDA-3	87.7	71.8	0.44 ± 0.06	65.1 ± 2.2

^a^ The crystallinity index (CrI) of the chitosan samples was determined using Equation (1). The mechanical experiment for each sample was conducted in triplicate.

## Data Availability

The original contributions presented in this study are included in the article/Supplementary Materials. Further inquiries can be directed to the corresponding author.

## Supplementary Materials

The following supporting information can be downloaded at: https://www.mdpi.com/article/10.3390/polym18161932/s1, Figure S1. FTIR spectra for the CS, CPDA-1, CPDA-2, and CPDA-3. Figure S2. The size of PDA particles in CPDA-1, CPDA-2, and CPDA-3. Figure S3. The HeLa cells in vitro cytotoxicity tests on different hydrogel samples. Figure S4. DPPH Radical scavenging activity of CPDA-1 (a), CPDA-2 (b), and CPDA-3 (c).
