# Supplementary material for: In Situ Construction of High Strength Chitosan/Polydopamine Hydrogel in Alkali System with Potential Radical Scavenging and Bacterial Inhibition
Source: Polymers (Basel). 2026 Aug 7;18(16):1932. doi: 10.3390/polym18161932 (PMC13516051; doi:10.3390/polym18161932)
Supplement: Supplementary file 1 [file polymers-18-01932-s001.zip › polymers-4417322-supplementary.pdf]

Supporting Information

In Situ Construction of High Strength Chitosan/Polydopamine Hydrogel in Alkali System with Potential Radical Scavenging and Bacterial Inhibition

Zhiwei Jiang [1,2,3,*,†], Xiang Xiao [3,†], Meng Yu [2], Qiyang Wang [4,*] and Ping Fang [3]

1 China School of Environmental Science and Engineering, Sun Yat-Sen University, Guangzhou 510275, China
2 Key Laboratory of Advanced Energy Materials Chemistry (Ministry of Education), Nankai University, Tianjin 300071, China
3 The Key Laboratory of Water and Air Pollution Control of Guangdong Province, Guangzhou 510655, China
4 School of Chemistry and Chemical Engineering, Anhui University, Hefei 230601, China
* Correspondence: jiangzhw8@mail.sysu.edu.cn (Z.J.); qywang@ahu.edu.cn (Q.W.)
† These authors contribute equally to this work.

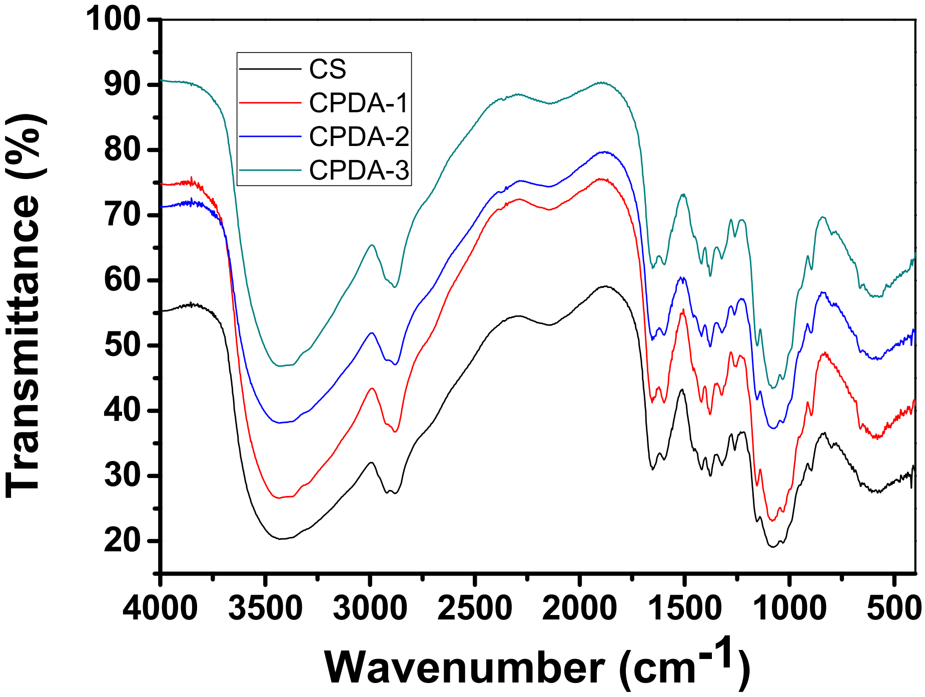

Figure S1. FTIR spectra for the CS, CPDA-1, CPDA-2, and CPDA-3.

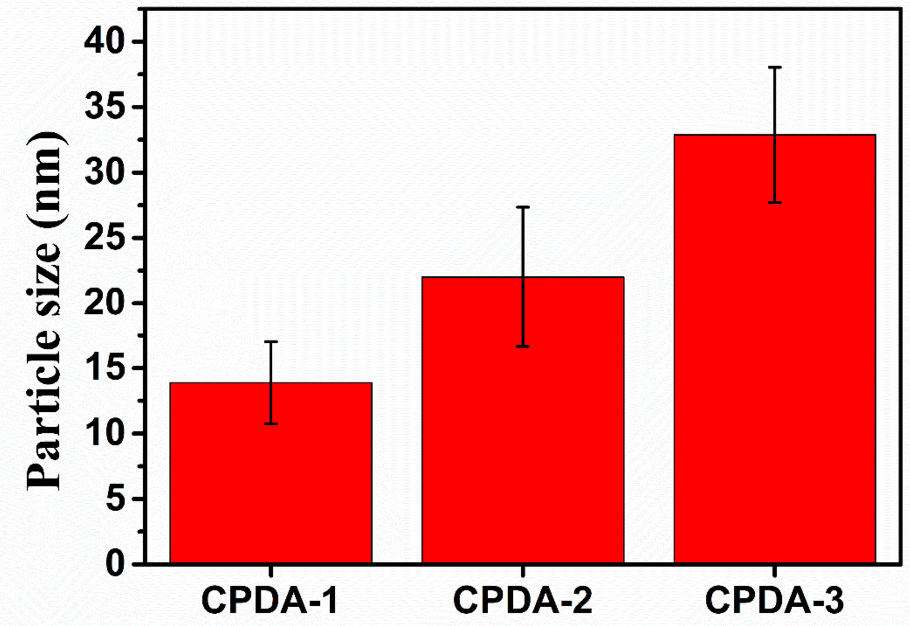

Figure S2. The size of PDA particles in CPDA-1, CPDA-2, and CPDA-3.

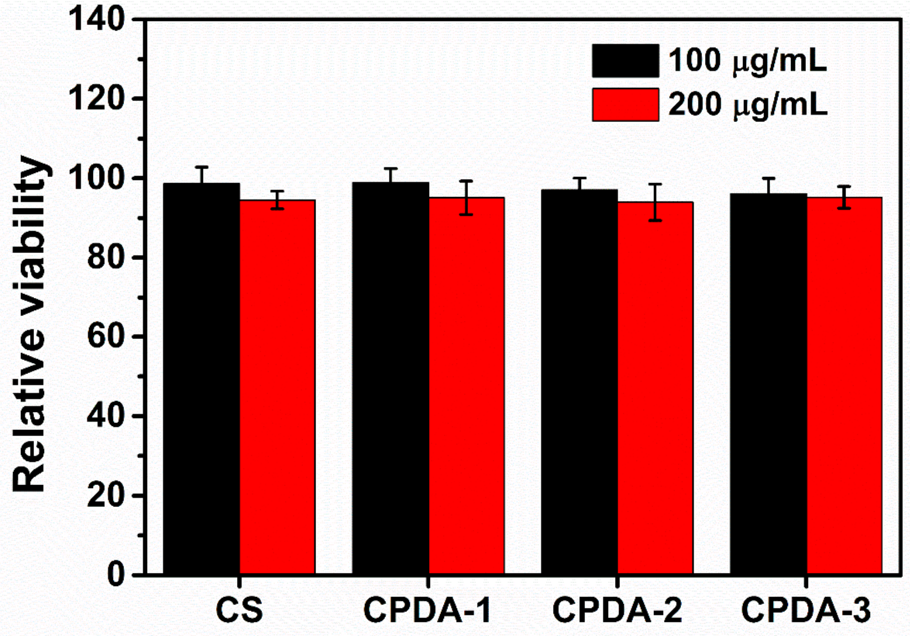

Figure S3. The HeLa cells *in vitro* cytotoxicity tests on different hydrogel samples.

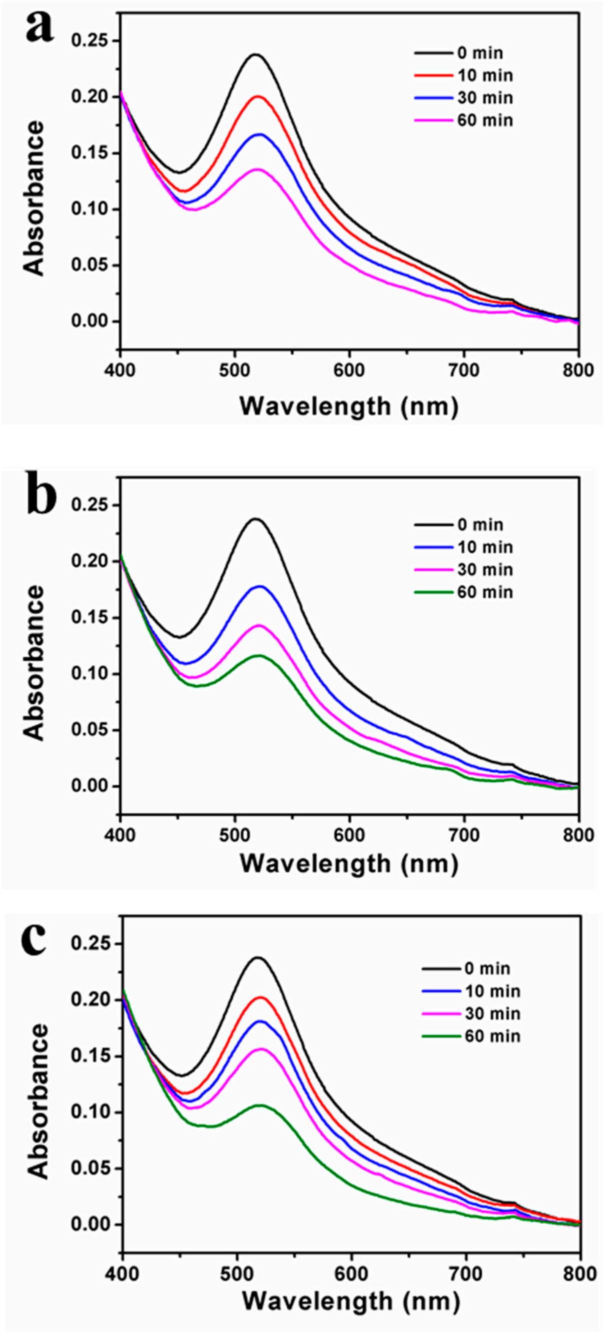

Figure S4. DPPH Radical scavenging activity of CPDA-1 (a), CPDA-2 (b), and CPDA-3 (c).
